# Supplementary material for: Hybrid Models and Biological Model Reduction with PyDSTool
Source: PLoS Comput Biol. 2012 Aug 9;8(8):e1002628. doi: 10.1371/journal.pcbi.1002628 (PMC3415397; doi:10.1371/journal.pcbi.1002628)
Supplement: Text S4 — Complete source code for the PyDSTool package (version 0.88.120504). Includes API documentation and help files linking to web pages. This file is identical to the current public release on Sourceforge.net. (ZIP) [file pcbi.1002628.s004.zip › PyDSTool/html/PyDSTool.Toolbox.optimizers.line_search.quadratic_interpolation-pysrc.html]

xml version="1.0" encoding="ascii"?


PyDSTool.Toolbox.optimizers.line\_search.quadratic\_interpolation


| Home | Trees | Indices | Help | PyDSTool |
| --- | --- | --- | --- | --- |

Package PyDSTool ::
Package Toolbox ::
Package optimizers ::
Package line\_search ::
Module quadratic\_interpolation

[hide private]

[frames] | no frames]

# Source Code for Module PyDSTool.Toolbox.optimizers.line\_search.quadratic\_interpolation

```
 1   
 2  # Matthieu Brucher 
 3  # Last Change : 2007-08-26 19:51 
 4   
 5  """ 
 6  Line Search with the quadratic interpolation method with the computation of the gradient of the function 
 7  """ 
 8   
 9  import math 
10  import numpy 
11   


12 -class QuadraticInterpolationSearch(object):


13    """ 
14    Line Search with the quadratic interpolation when the gradient of the function is provided 
15    """ 


16 -  def __init__(self, min_alpha_step, alpha_step = 1., **kwargs):


17      """ 
18      Needs to have : 
19        - a minimum step size (min_alpha_step) 
20      Can have : 
21        - a step modifier, a factor to modulate the step (alpha_step = 1.) 
22      """ 
23      self.minStepSize = min_alpha_step 
24      self.stepSize = alpha_step

25   


26 -  def __call__(self, origin, function, state, **kwargs):


27      """ 
28      Returns a good candidate 
29      Parameters : 
30        - origin is the origin of the search 
31        - function is the function to minimize 
32        - state is the state of the optimizer 
33      """ 
34      direction = state['direction'] 
35      ak = 0. 
36      if 'initial_alpha_step' in state: 
37        bk = state['initial_alpha_step'] 
38      else: 
39        bk = self.stepSize 
40      v_bk = function(origin + bk * direction) 
41   
42      while abs(bk - ak) > self.minStepSize: 
43        v_ak = function(origin + ak * direction) 
44        g_ak = numpy.dot(function.gradient(origin + ak * direction), direction) 
45        ck = ak - .5 * (ak - bk) * g_ak / (g_ak - (v_ak - v_bk) / (ak - bk)) 
46        v_ck = function(origin + ck * direction) 
47   
48        bk = ak 
49        ak = ck 
50        v_bk = v_ak 
51        v_ak = v_ck 
52   
53      state['alpha_step'] = ck 
54      return origin + ck * direction

55
```

  


| Home | Trees | Indices | Help | PyDSTool |
| --- | --- | --- | --- | --- |

|  |  |
| --- | --- |
| Generated by Epydoc 3.0.1 on Fri May 4 15:24:18 2012 | http://epydoc.sourceforge.net |
